# Enhancing effectiveness of capillary electrophoresis as an analytical tool in the supramolecular acidity modification

**DOI:** 10.1007/s00216-017-0305-y

**Published:** 2017-03-24

**Authors:** Paweł Mateusz Nowak, Michał Woźniakiewicz, Magdalena Janus, Paweł Kościelniak

**Affiliations:** Faculty of Chemistry, Department of Analytical Chemistry, Jagiellonian University in Kraków, Ingardena 3, 30-060 Kraków, Poland

**Keywords:** Capillary electrophoresis, Capillary coating, Cyclodextrins, Host-guest complexes, Joule heating, p*K*_a_ shift

## Abstract

A strategic modification of acidity (p*K*_a_ values) by the non-covalent host-guest interactions is one of the most promising concepts in current supramolecular chemistry. This work is aimed at enhancing the effectiveness of capillary electrophoresis (CE) in determination of p*K*_a_ shifts caused by such interactions and their thermal dependencies crucial in a deep thermodynamic description. We show how to (i) minimize the systematic errors related to Joule heating, (ii) minimize the influence of a voltage ramp time, (iii) speed up p*K*_a_ shift identification and estimation, (iv) interpret thermal effects related to two overlapped dynamic equilibria, and (v) determine p*K*_a_ shifts by an alternative spectrophotometric method (CE-DAD). The proposed solutions were implemented to examine the supramolecular p*K*_a_ shifts of several coumarin derivatives, caused by a variety of structurally different cyclodextrins. It was revealed that a specific host substitution pattern determines the magnitude of apparent p*K*_a_ shifts. Accordingly, heptakis(2,6-di-*O*-methyl)-β-cyclodextrin induces the much stronger shifts than both non-methylated-β-cyclodextrin and heptakis(2,3,6-tri-*O*-methyl)-β-cyclodextrin applied at the same concentration. We also show that insofar as the complexation of 4-hydroxycoumarin and its derivative (coumatetralyl) are similarly exothermic, the thermal effects accompanying the deprotonation process are remarkably different for both molecules. The p*K*_a_ shift induced by complexation with calixarene was also for the first time determined by a CE method. These observations throw a new light on the background of acidity modification and confirm the applicability of CE as an analytical tool.

**Electronic supplementary material:**

The online version of this article (doi:10.1007/s00216-017-0305-y) contains supplementary material, which is available to authorized users.

## Introduction

Acidity, usually expressed by a logarithmic equivalent of acid dissociation constant—p*K*_a_, is one of the basic physicochemical properties of the ionizable molecules [1]. It determines their solubility in aqueous environment, lipophilicity and membrane permeability, ability to ionic and hydrophobic interactions, activity and functionality in biological systems (including endo- and exo-genic compounds), and particular behaviors in analytical systems, e.g., retention on column in chromatography or migration time in electrophoresis. The idea to modify p*K*_a_ using the non-covalent interactions, known as a supramolecular acidity (p*K*_a_) tuning, is a potential way to enhance bioavailability and therapeutic activity of drugs [1–3], to modulate physicochemical properties of dyes [4, 5], to create sophisticated catalysis systems [6], and to develop more efficient analytical methods [7]. The recent studies showed that the supramolecular host-guest interactions involving the macrocyclic hosts, like cyclodextrins (CDs), calixarenes, and cucurbiturils, may induce p*K*_a_ shifts in both directions and of a diverse magnitude, reaching +1.5 pH unit for CDs, +2.4 for calixarenes, and even above +5 units for cucurbiturils [1, 7, 8]. However, our current knowledge on these effects is still scarce and limited, and our ability to predict the magnitude of p*K*_a_ shifts is weak. Hence, one of the most promising directions of research in this field is the analysis of novel p*K*_a_ modification systems and thermodynamic investigations focusing on the basic molecular forces governing p*K*_a_ shifts, expressed quantitatively by the enthalpic and entropic factors [8–10].

Capillary electrophoresis (CE) is a powerful separation technique characterized by a huge resolving power, high automation degree, and, most importantly, very low consumption of sample materials and buffers, in the order of micro- and even nano-liters. Among various analytical and bioanalytical applications, CE is broadly utilized in determination of p*K*_a_ values [11–14]. It is done by plotting the relation between electrophoretic mobility, dependent on ionization degree, and pH, and finding p*K*_a_ as an inflection point of the obtained sigmoidal curve. Electrophoretic mobility can be calculated as follows:

1 $$ {\mu}_{\mathrm{ep}}=\frac{L_{\mathrm{tot}}\ {L}_{\mathrm{eff}}}{U_{\mathrm{nom}}}\bullet \left(\frac{1}{t_{\mathrm{tot}}}-\frac{1}{t_{\mathrm{eof}}}\right) $$

where *μ*_ep_ is the electrophoretic mobility, *L*_tot_ and *L*_eff_ are the total and effective capillary lengths (m), *U*_nom_ is the nominal (programmed) separation voltage (kV), *t*_tot_ is the total (observed) migration time of the analyte (min), and *t*_eof_ is the time measured for the neutral marker of electroosmotic flow (EOF).

For a monoprotic acid, p*K*_a_ can be obtained from the following equation:

2 $$ {\mu}_{\mathrm{ep}}=\left[\frac{10^{-{\mathrm{p} K}_{\mathrm{a}}}}{10^{-{\mathrm{p} K}_{\mathrm{a}}}+{10}^{-\mathrm{pH}}}\right]\bullet {\mu}_{{\mathrm{A}}^{-}} $$

where *μ*_A_^−^ is the electrophoretic mobility of the anion created upon deprotonation and p*K*_a_ is a fitting parameter (pH at the inflection point).

**Fig. 1:**
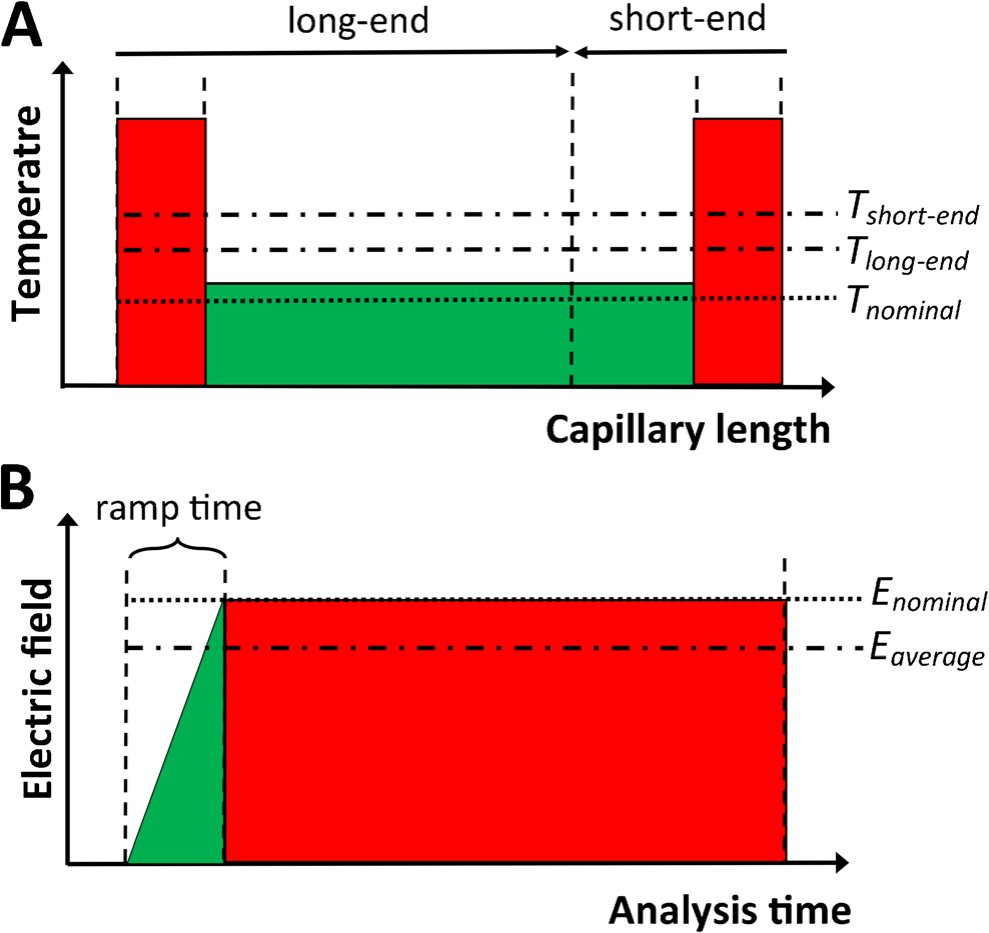
Schematic illustration of **a** the profile of actual temperature along capillary, with an indication of the average temperatures for two injection modes (long-end and short-end): *T*_long-end_ and *T*_short-end_, respectively, and nominal temperature set up in a software—*T*_nominal_ and **b** the ramping effect, with an indication of the average and nominal electric field strength, *E*_average_ and *E*_nominal_, respectively

In consequence, CE characterized by a prominent cost-effectiveness seems also to be a valuable analytical tool for examination of the supramolecular p*K*_a_ modification systems, often involving expensive macrocyclic molecules used as the p*K*_a_ shift inducers. In addition, due to the ability of modern CE instruments to control temperature in a broad range, normally between 15 and 60 °C, CE may be useful in examination of thermal dependency of p*K*_a_ values and in determination of the standard dissociation enthalpy (Δ*H*°) and entropy (Δ*S*°). Nonetheless, apart from advantages, utilization of CE for those purposes is not free from potential limitations. The important issues are as follows:

(i) Systematic errors attributed to the uncontrolled rise of temperature inside capillary caused by Joule heating generation and insufficient cooling [15, 16], especially in the inlet/outlet capillary sections devoid of an active cooling (see Fig. 1a).
(ii) Systematic errors caused by a ramping effect, i.e., a gradual increase of voltage at the beginning of separation, lasting around 0.1–0.2 min, which causes a deviation of the average electric field strength from its nominal value used in Eq. 1 (see Fig. 1b).
(iii) Relatively long analysis time, taking into account the fact that to determine one p*K*_a_ value using a classical method electrophoretic mobility should be determined in at least 5–6 buffers of different pH, whereas a single CE run lasts typically between 5 and 30 min (including capillary rinsing and conditioning) and each one should be done at least in triplicate to minimize random errors.
(iv) Difficulty to reach a total complexation degree in host-guest systems, as a result the p*K*_a_ values measured at a given host concentration are only apparent ones, i.e., they do not characterize a host-guest complex but a mixed population of the complexed and free molecules, and thus they depend on two equilibria: deprotonation and complexation. The knowledge on the apparent p*K*_a_ shifts is however still desirable because the total complexation degree is rarely met in real experiments using aqueous solutions of hosts, and the apparent p*K*_a_ values characterize a true behavior of molecules, e.g., in the CD-separation systems. Nonetheless, in such a case, thermodynamic interpretations are hampered.
(v) Problematic use of the ionized hosts, e.g., sulphated CDs and calixarenes, introducing additional charge that affects electrophoretic mobility and intrudes in the relation between ionization of guest and pH.

**Fig. 2:**
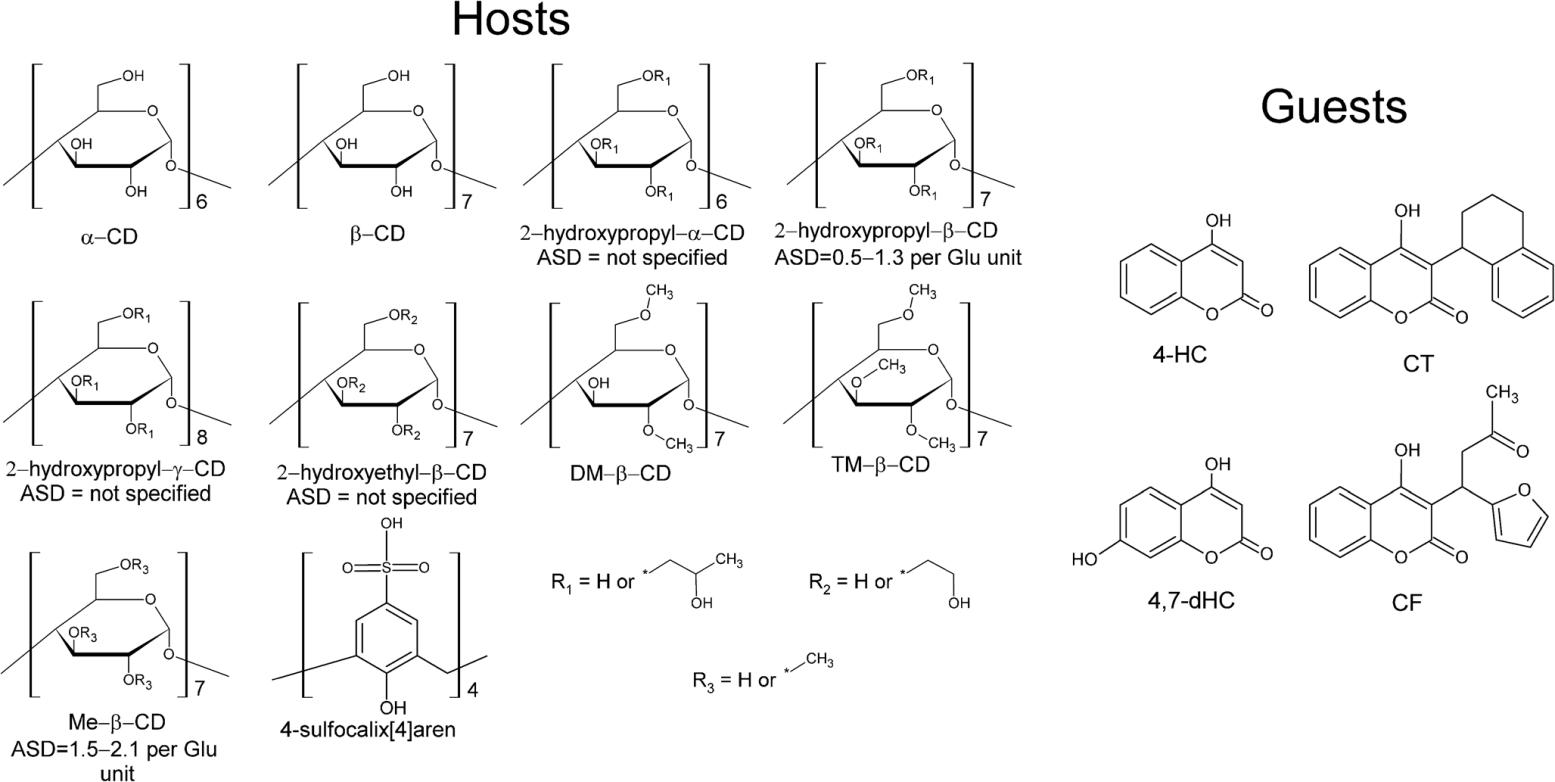
The structures of guest and host molecules used in the experiment. *ASD* average substitution degree

The aim of this work was to tackle these general issues and to enhance effectiveness of CE as an analytical method for determination of p*K*_a_ values and their shifts in the supramolecular host-guest systems. We show that minimization of the aforementioned problems is possible via (i) utilization of a proper correction strategy basing on the estimation of an actual temperature inside capillary, (ii) concurrent correction of the ramping effect by a simple recalculation, (iii) utilization of a two-values method (TVM) restricted only to two electrophoretic mobility values needed to detect and estimate p*K*_a_ shift, (iv) specific data handling/analysis that provides an independent insight into two equilibria, and (v) measurement of absorption spectra instead of mobilities in the case of ionizable hosts. Furthermore, we examined p*K*_a_ shifts of several coumarin derivatives: 4-hydroxycoumarin (4-HC), 4,7-dihydroxycoumarin (4,7-dHC), coumatetralyl (CT), and coumafuryl (CF), caused by a variety of neutral CDs: α-CD, β-CD, 2-hydroxypropyl-α-CD, 2-hydroxypropyl-β-CD, 2-hydroxypropyl-γ-CD, 2-hydroxyethyl-β-CD, methyl-β-CD (Me-β-CD), heptakis(2,6-di-*O*-methyl)-β-cyclodextrin (DM-β-CD), and heptakis(2,3,6-tri-*O*-methyl)-β-cyclodextrin (TM-β-CD), in some cases providing an insight into the enthalpic and entropic contributions. We also estimated p*K*_a_ shift of 4-HC caused by 4-sulfo-calix[4]arene, using an alternative CE-DAD method. The corresponding structures are shown in Fig. 2. The obtained outcomes demonstrate a key role of host substitution and a strict correlation between the thermal effects accompanying deprotonation and the structure of guests.

## Materials and methods

Four coumarin derivatives—4-HC, 4,7-dHC, CT, and CF—used as the analytes (guests), and ten macrocyclic hosts—α-CD, β-CD, 2-hydroxypropyl-α-CD, 2-hydroxypropyl-β-CD, 2-hydroxypropyl-γ-CD, 2-hydroxyethyl-β-CD, Me-β-CD, DM-β-CD, TM-β-CD, and 4-sulfo-calix[4]arene—were supplied by Sigma-Aldrich (St. Louis, MO, USA). Dimethyl sulfoxide (DMSO) was used as the EOF marker (Sigma-Aldrich).

The experiments were performed with a P/ACE MDQ Capillary Electrophoresis (CE) System (Beckman Coulter, Brea, CA, USA) equipped with a diode array detector. A polyamine-coated eCAP™ capillary was used (Beckman Coulter); it was of 60 cm total length, 50 cm effective length, and 50 μm internal diameter. The UV-vis absorption spectra were collected between 200 and 600 nm. Two hundred nanometers was the analytical wavelength for measuring electrophoretic mobility. The sample trays and capillaries were conditioned at various temperatures, between 15 and 55 °C. All aqueous solutions were prepared using a deionized water (MilliQ, Merck-Millipore Billerica, MA, USA), filtered through a 0.45-μm regenerated cellulose membrane and then degassed by sonication and centrifugation. The rinsing of capillaries between the runs was done by applying a pressure of 137.9 kPa (20 psi). The capillary, between the runs, was rinsed with 0.1 M amine regenerator solution™ (Beckman Coulter) for 2 min and with the background electrolyte (BGE) for 4 min. Prior the first use of the capillary on a working day a rinse with deionized water for 5 min, 0.1 M amine regenerator solution™ for 10 min, and BGE for 10 min was applied. For a fresh capillary conditioning, the same rinsing sequence was used but the duration of each step was doubled.

All BGE solutions were of a 50-mM ionic strength, except with BGEs containing 4-sulfo-calix[4]arene where it was increased to 92.5 mM. The final concentration of each CD in BGE was 10 mM and 4-sulfo-calix[4]arene 5 mM. The exact composition and pH values are presented in the Electronic Supplementary Material (ESM). Sample injection was done hydrodynamically, applying the forward pressure of 3.45 kPa (0.5 psi) for 5 s. 4-HC and CT were used in a final concentration of 100 μg/mL, 4,7-dHC and CF 33 μg/mL, and DMSO 0.2% (*v*/*v*), all dissolved in BGE. Electrophoretic analysis was performed with a separation voltage of 30 kV, using a reverse polarity (cathode at inlet), and a 0.17-min voltage ramp time. All measurements were done in triplicate. The methodology of an actual temperature estimation by a simplified universal method for electrolyte temperature determination (SUMET) [17] and the Van’t Hoff model used to determine the enthalpic and entropic factors are described in ESM.

## Results and discussion

### A preliminary capillary test

**Fig. 3:**
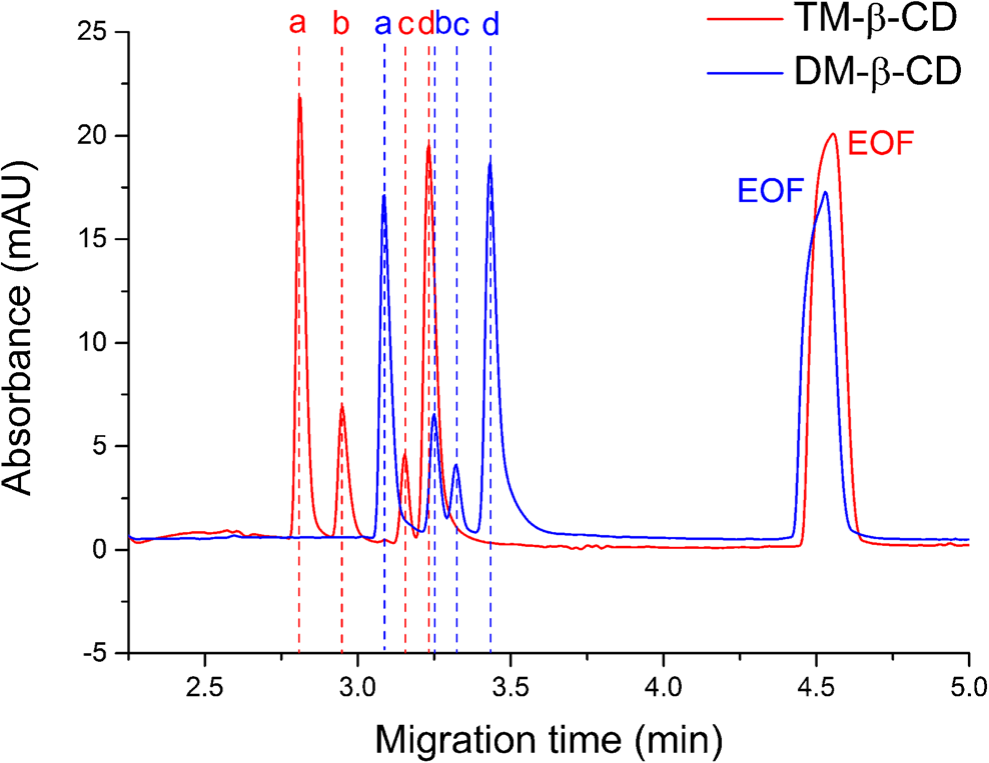
Electropherograms obtained for the sample containing the totally ionized analytes: 4-HC (*a*), 4,7-dHC (*b*), CF (*c*), CT (*d*), and EOF marker (DMSO), in BGE containing 10 mM DM-β-CD or TM-β-CD (pH 8.0, temperature 25 °C). The similar position of the EOF peaks indicates similar values of electroosmotic mobility, whereas the significant shifts noted for the analytes indicate different electrophoretic mobility values stemming from a different complexation degree (affinity) for two structurally similar CDs

At the beginning, the eCAP™ amine capillary was tested in terms of repeatability estimated for electrophoretic mobilities, a crucial issue in minimization of the random uncertainty of p*K*_a_ values. This capillary occurred to provide very stable conditions, what was evidenced by the very low RSD (%) values, mostly much below 1.0%. It was shown and discussed in the ESM. In addition, the generation of a reverse EOF, i.e., in the opposite direction than in the bare silica capillary, allowed one to detect all analytes prior to the EOF marker making the whole analysis much faster (see electropherogram in Fig. 3).

### Two-values method for faster p*K*_a_ shift identification/estimation

It was recently postulated by our group that a routine method used for p*K*_a_ determination, basing on the multiple measurements of electrophoretic mobility in a broad pH range and fitting the sigmoidal-shape function (Eq. 2), can be replaced by a much faster method using only two electrophoretic mobility values [18]. For a monoprotic acid, we can write the following:

3 $$ {\mathrm{p} K}_{\mathrm{a}}=\mathrm{pH}+ \log \frac{\mu_{{\mathrm{A}}^{-}}-{\mu}_{\mathrm{ep}}}{\mu_{\mathrm{ep}}} $$

where *μ*_ep_ is the electrophoretic mobility of the partially ionized form reached at a given pH, and *μ*_A_− is the electrophoretic mobility of anion (totally ionized form).

This alternative approach, denoted as a two-values method (TVM), requires the application of only two buffers, ensuring the partial and total ionization, respectively. TVM was proven to give consistent results with the classical method with the mean deviation of p*K*_a_ much below 0.20 pH unit, providing 2–3 times better time- and cost-effectiveness [18]. In this work, TVM was for the first time applied to detect and estimate the supramolecular p*K*_a_ shifts. The outcomes obtained at the temperature of 25 °C for four analytes (guests) and nine hosts are presented in Table 1.

**Table 1:** The values of p*K*_a_ and the respective p*K*_a_ shifts obtained with the TVM-based and the classical approaches

System	p*K*_a_ value				p*K*_a_ shift			
	4-HC	4,7-diHC	CT	CF	4-HC	4,7-diHC	CT	CF
reference^a^	4.13	4.38	4.25	4.16	–	–	–	–
α-CD	4.15	4.36	4.10	3.74	+0.02	−0.02	−0.15	−0.43
β-CD	4.70	4.52	4.82	4.18	+0.57	+0.13	+0.57	+0.02
2HP-α-CD	4.10	4.36	4.14	3.75	−0.03	−0.03	−0.11	−0.41
2HP-β-CD	4.68	4.74	5.00	4.81	+0.55	+0.36	+0.76	+0.65
2HP-γ-CD	4.37	4.64	4.66	4.44	+0.24	+0.26	+0.41	+0.27
2HE-β-CD	4.62	4.67	4.88	4.60	+0.49	+0.29	+0.63	+0.43
Me-β-CD	4.80	4.80	4.97	4.67	+0.67	+0.41	+0.72	+0.51
DM-β-CD	5.08	5.00	5.03	5.20	+0.95	+0.62	+0.78	+1.04
TM-β-CD	4.54	4.43	4.29	4.76	+0.41	+0.05	+0.04	+0.60
DM-β-CD	5.19^b^	–	5.18^b^	–	+1.12^b^	–	+0.86^b^	–
TM-β-CD	4.61^b^	–	4.47^b^	–	+0.54^b^	–	+0.15^b^	–

The mobility of the partially ionized forms (TVM) were measured at pH close to 4.6. The standard error (SD) obtained for TVM was below 0.15 pH unit in each case

^a^A host-free system

^b^The values obtained with the classical method (nonlinear function fitting)

The observed p*K*_a_ shifts are diverse, reach up to +1.04 pH unit, and strongly depend on the structure of host. It was more deeply discussed in the next section (“Thermodynamic analysis”). To check reliability of these results, the p*K*_a_ shifts of 4-HC and CT in two systems—DM-β-CD and TM-β-CD—were also determined using the classical approach based on a nonlinear function fitting (see Table 1). For these two CDs, a discernible difference in the magnitude of p*K*_a_ shifts was revealed by TVM. The obtained reference shifts are slightly higher, averagely by 0.13 pH unit. Nevertheless, an overall magnitude remains similar and the noticeable difference between both CDs is also maintained. It proves that TVM may be useful in a fast screening/detection of p*K*_a_ shifts in the supramolecular systems and that it enables their estimation with some margin of error. Due to a simplified nature, the use of an “estimation” term seems better in regard to TVM than “determination.” It is also worth emphasizing that the concurrent use of the amine capillary and TVM seems to be beneficial and advisable, since both reduce p*K*_a_ estimation time and the amine capillary may also provide a stable EOF [18, 19]. It gives the opportunity to examine many novel p*K*_a_ modification systems and to minimize the related costs and analysis time.

### Double correction strategy for minimization of the systematic errors

An accurate measurement of electrophoretic mobility is of a great importance for reliable p*K*_a_ determination. However, as it was mentioned before, it is a routine practice that two important effects able to introduce systematic errors to mobility value are not taken into consideration: the voltage ramping effect and the rise of temperature due to the Joule heating (Fig. 1). To the best of our knowledge, the influence of these effects on the accuracy of p*K*_a_ determination has never been examined. Herein, we propose an original approach, a universal double correction strategy (DCS) which allows one to estimate this impact and to minimize the inaccuracy of p*K*_a_ values and the other important thermodynamic parameters, standard dissociation enthalpy (Δ*H*°), and entropy (Δ*S*°), dependent on a thermal dependency of p*K*_a_ values. We implemented DCS in the classical methodology of p*K*_a_ determination basing on a nonlinear function fitting, to enhance its accurateness and reliability. DCS comprises several steps:

Firstly, the ramping effect is corrected using the following equations:

4 $$ {U}_{\mathrm{av}}={U}_{\mathrm{nom}}\left(\frac{t_{\mathrm{tot}}-0.5{t}_{\mathrm{ramp}}}{t_{\mathrm{tot}}}\right) $$

where *U*_av_ is the average (ramping-corrected) separation voltage, *t*_tot_ is the total migration time of the analyte, and *t*_ramp_ is the voltage ramp time set up in a software, normally between 0.1 and 0.2 min. This equation follows from the profile of electric field shown in Fig. 1b.

It is to be noted that *U*_av_ is normally different for the analyte and EOF marker because they differ in migration time, and thus the impact of voltage ramp time is not the same for both compounds. To respect this fact, electrophoretic mobility should be calculated as follows:

5 $$ {\mu}_{\mathrm{ep}\left(\mathrm{ramp}\right)}={\mu}_{\mathrm{app}\left(\mathrm{ramp}\right)}-{\mu}_{\mathrm{eof}\left(\mathrm{ramp}\right)} $$

6 $$ {\mu}_{\mathrm{ep}\left(\mathrm{ramp}\right)}=\frac{L_{\mathrm{tot}}\ {L}_{\mathrm{eff}}}{U_{\mathrm{nom}}\ {t}_{\mathrm{tot}}}\left(\frac{t_{\mathrm{tot}}}{t_{\mathrm{tot}}-0.5{t}_{\mathrm{ramp}}}\right)-\frac{L_{\mathrm{tot}}\ {L}_{\mathrm{eff}}}{U_{\mathrm{nom}}\ {t}_{\mathrm{eof}}}\left(\frac{t_{\mathrm{eof}}}{t_{\mathrm{eof}}-0.5{t}_{\mathrm{ramp}}}\right) $$

and after simplification,

7 $$ {\mu}_{\mathrm{ep}\left(\mathrm{ramp}\right)}=\frac{L_{\mathrm{tot}}\ {L}_{\mathrm{eff}}}{U_{\mathrm{nom}}\ \left({t}_{\mathrm{tot}}-0.5{t}_{\mathrm{ramp}}\right)}-\frac{L_{\mathrm{tot}}\ {L}_{\mathrm{eff}}}{U_{\mathrm{nom}}\ \left({t}_{\mathrm{eof}}-0.5{t}_{\mathrm{ramp}}\right)} $$

where *μ*_ep(ramp)_ is the ramping-corrected electrophoretic mobility, *μ*_app(ramp)_ is the ramping-corrected apparent (observed) mobility, and *μ*_eof(ramp)_ is the ramping-corrected electroosmotic mobility (compare with Eq. 1). The impact of ramping effect, i.e., the corresponding error of p*K*_a_ value, can be estimated by comparing p*K*_a_ values obtained using two different methods for electrophoretic mobility determination, based on Eqs. 1 and 7, respectively.

The second step is the correction of p*K*_a_ values in regard to temperature rise. Temperature elevation entails two major effects: drop in viscosity and, for some molecules, temperature-dependent change of the ionization degree (p*K*_a_), both affecting electrophoretic mobility values. It is also noticeable that, in theory, the buffers used in p*K*_a_ determination should be of the same ionic strength and exhibit different pH; therefore, they are normally prepared using the varied acids and bases. Thus, they differ to some extent in conductivity and, in consequence, in the values of current measured upon application of voltage. Therefore, temperature rise is never the same at all pH values and the procedure used to determine p*K*_a_ is intrinsically biased by a double error (rise in temperature and inequality of this rise at various pH values).

**Fig. 4:**
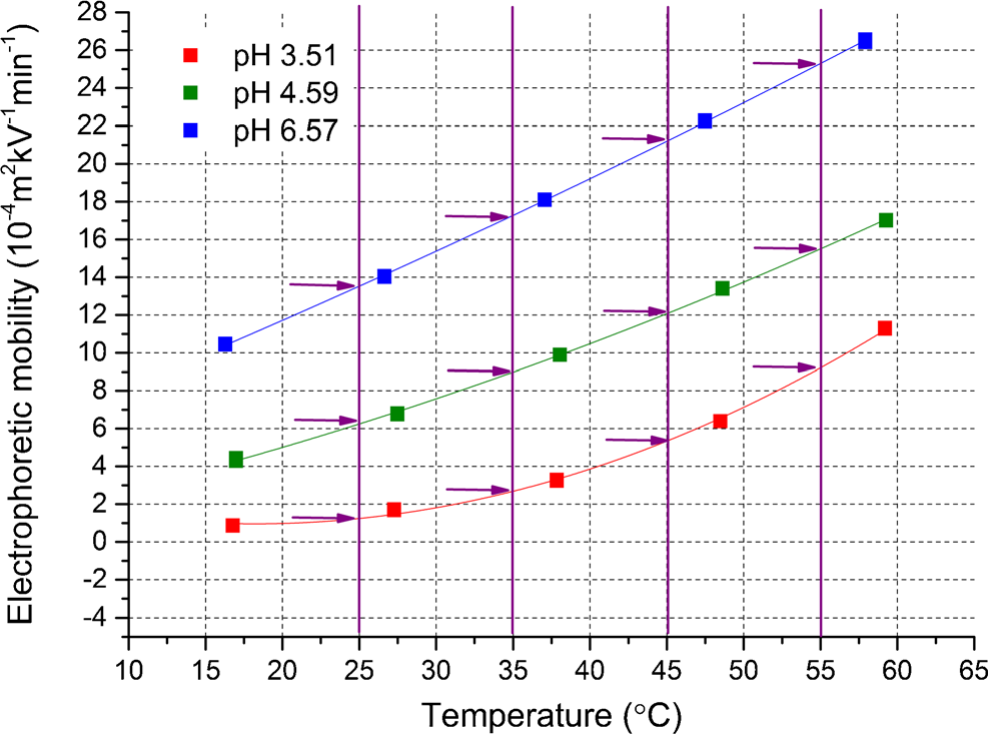
Illustration of a temperature increase-related correction of electrophoretic mobilities; the procedure is as follows: (1) electrophoretic mobilities (corrected previously in regard to the ramping effect) are measured at various coolant temperatures, and at various pH values; (2) the actual (real, effective) temperature values are estimated; (3) the obtained mobilities are plotted versus the actual temperature and fitted with a proper model (in this case by the second-degree polynomial functions); (4) electrophoretic mobility values corresponding to the given temperature of interest (in this case, 25, 35, 45 and 55 °C) are read from the model functions (see the *arrows* on the graph), and then they are used to calculate p*K*_a_ values. Presentation of data is restricted to 3 pH values and 4 temperatures of interest for a better clearness

We propose that this problem may be tackled by the estimation of an actual temperature reached in the given buffer (*T*_actual_) at various temperatures of coolant and by the subsequent plotting of the obtained electrophoretic mobilities versus *T*_actual_. Then, after function fitting, by inter- or extrapolation, we can read electrophoretic mobility values corresponding to the chosen temperatures of interest, the same for all buffering systems (see Fig. 4). It allows one to determine a correct p*K*_a_ value, unbiased by Joule heating. This strategy is an extension of the correction approach proposed by us recently for correcting electrophoretic mobilities [20].

The estimation of an actual temperature is possible via a simplified universal method for electrolyte temperature determination (SUMET), reported by Krylov [17]. SUMET enables estimation of temperature rise separately for the non-cooled and cooled capillary parts, using only the values of current measured during CE run and some tabulated parameters, determined for the given capillary dimensions. It was assumed by us that the amine and bare capillaries of the same length and internal diameter are characterized by the same heat transport efficiency. The detailed description can be found in the ESM. We suggest herein to use SUMET for both capillary sections and to assess an actual temperature (to which calculated mobility refers) as a weighted average as described by Eq. 8.

8 $$ {T}_{\mathrm{actual}}={T}_{\mathrm{inlet}}\left(\frac{L_{\mathrm{inlet}}}{L_{\mathrm{eff}}}\right)+{T}_{\mathrm{core}}\left(\frac{L_{\mathrm{eff}}-{L}_{\mathrm{inlet}}}{L_{\mathrm{eff}}}\right) $$

where *T*_inlet_ is the local temperature in the inlet non-thermostated capillary section obtained from SUMET, *T*_core_ is the local temperature in the core thermostated capillary section obtained from SUMET, and *L*_inlet_ is the length of inlet section (4.0 cm in the present study). In this approach, the weights are defined by the respective lengths of the particular capillary sections; however, this is an arbitrary choice and the other weights could also be used, e.g., time-related ones, though their calculation would more complicated.

The obtained values of actual temperature are presented in the ESM. In Table 2, we present the non-corrected, ramping-corrected, and totally corrected p*K*_a_ values obtained for 4-HC and CT, together with the corresponding enthalpic (Δ*H*°) and entropic (Δ*S*°) terms obtained from the Van’t Hoff model (see the ESM for more details).

**Table 2:** Comparison of the non-corrected, ramping-corrected, and fully corrected values of acid dissociation constant (p*K*_a_), standard dissociation enthalpy (Δ*H*°), and standard dissociation entropy (Δ*S*°) obtained for the two analytes (4-HC and CT) in the two host-guest systems (10 mM DM-β-CD and TM-β-CD)

Temperature (°C)	Non-corrected	Ramping corrected	Fully corrected
4-HC + DM-β-CD			
15	5.33 (0.03)	5.31 (0.03)	5.33 (0.02)
25	5.19 (0.03)	5.17 (0.03)	5.23 (0.02)
35	5.08 (0.04)	5.05 (0.04)	5.12 (0.04)
45	4.97 (0.03)	4.94 (0.04)	5.00 (0.03)
55	4.84 (0.04)	4.80 (0.04)	4.88 (0.04)
Δ*H*° (kJ mol^−1^)	21.7 (0.6)	22.6 (0.7)	20.4 (0.9)
ΔS°(J mol^−1^ K^−1^)	−3.2 (0.2)	−2.8 (0.3)	−3.8 (0.4)
CT + DM-β-CD			
15	5.21 (0.02)	5.19 (0.03)	5.21 (0.02)
25	5.18 (0.03)	5.15 (0.03)	5.18 (0.03)
35	5.14 (0.03)	5.11 (0.03)	5.15 (0.03)
45	5.08 (0.03)	5.04 (0.04)	5.08 (0.03)
55	5.02 (0.04)	4.97 (0.04)	5.01 (0.04)
Δ*H*° (kJ mol^−1^)	8.6 (0.9)	9.9 (1.0)	9.0 (1.3)
Δ*S*°(J mol^−1^ K^−1^)	−8.4 (0.4)	−7.9 (0.4)	−8.3 (0.5)
4-HC + TM-β-CD			
15	4.73 (0.02)	4.71 (0.02)	4.77 (0.02)
25	4.61 (0.03)	4.59 (0.03)	4.64 (0.03)
35	4.51 (0.03)	4.48 (0.03)	4.54 (0.03)
45	4.44 (0.04)	4.39 (0.05)	4.46 (0.04)
55	4.38 (0.04)	4.32 (0.04)	4.38 (0.03)
Δ*H*° (kJ mol^−1^)	15.8 (1.0)	17.8 (0.7)	17.4 (0.7)
Δ*S*°(J mol^−1^ K^−1^)	−4.3 (0.4)	−3.4 (0.3)	−3.7 (0.3)
CT + TM-β-CD			
15	4.48 (0.03)	4.45 (0.03)	4.48 (0.03)
25	4.47 (0.03)	4.44 (0.03)	4.47 (0.02)
35	4.46 (0.03)	4.43 (0.03)	4.45 (0.03)
45	4.45 (0.04)	4.40 (0.04)	4.44 (0.03)
55	4.43 (0.04)	4.37 (0.05)	4.42 (0.02)
Δ*H*° (kJ mol^−1^)	2.2 (0.3)	3.6 (0.6)	2.7 (0.2)
Δ*S*°(J mol^−1^ K^−1^)	−9.4 (0.1)	−8.8 (0.3)	−9.2 (0.1)

The values in brackets are the standard errors estimated during the function fitting step

It turns out that both correction steps bring about the relatively small changes in the p*K*_a_ values, maximally 0.08 pH unit. It is noteworthy that the correction of the ramping effect always decreases p*K*_a_ values, whereas the correction of the temperature rise increases them and causes some compensatory effect. On that account, the total differences between the non-corrected and totally corrected values are even smaller, typically 0.02–0.05 pH unit. Despite the observed differences are small and comparable to the standard errors, the trends seen upon the first and second correction steps are clear, regular, and cannot be explained simply by a random variation. Taking into consideration the recent study that has revealed a significant influence of the Joule heating and ramping effects on the accuracy of electrophoretic mobilities [20], the resulting inaccuracy of the p*K*_a_ values studied in this work appears to be more tolerable. Most likely, this is due to the fact that the relative mobility error is similar at various pH values, and the position of inflection point in relation to pH scale is weakly affected.

On the other hand, the differences noted for the enthalpic and entropic factors are more considerable, in some cases, over 10%, but still the ramping- and temperature rise-related effects compensate for each other. The final differences in the enthalpic and entropic terms are also comparable to the standard errors, estimated for the Van’t Hoff model. These results imply that, for a routine determination of p*K*_a_ values under the “mild” conditions, correction of these effects may be unnecessary due to a need for conducting additional measurements. DCS requires at least two mobility measurements at two distinct temperatures, and thus, it is at least twice as time-consuming as the standard approach. It seems however recommended for determination of the enthalpic and entropic factors because in this case p*K*_a_ values are, by definition, determined at various temperatures, and the expected errors are larger. It is also worth highlighting that the errors could be more significant in other experimental setups, e.g., for the shorter capillaries, larger internal capillary diameters, and higher ionic and electric field strengths. DCS seems very useful for examination of these effects.

### Thermal effects in the overlapped equilibria

**Fig. 5:**
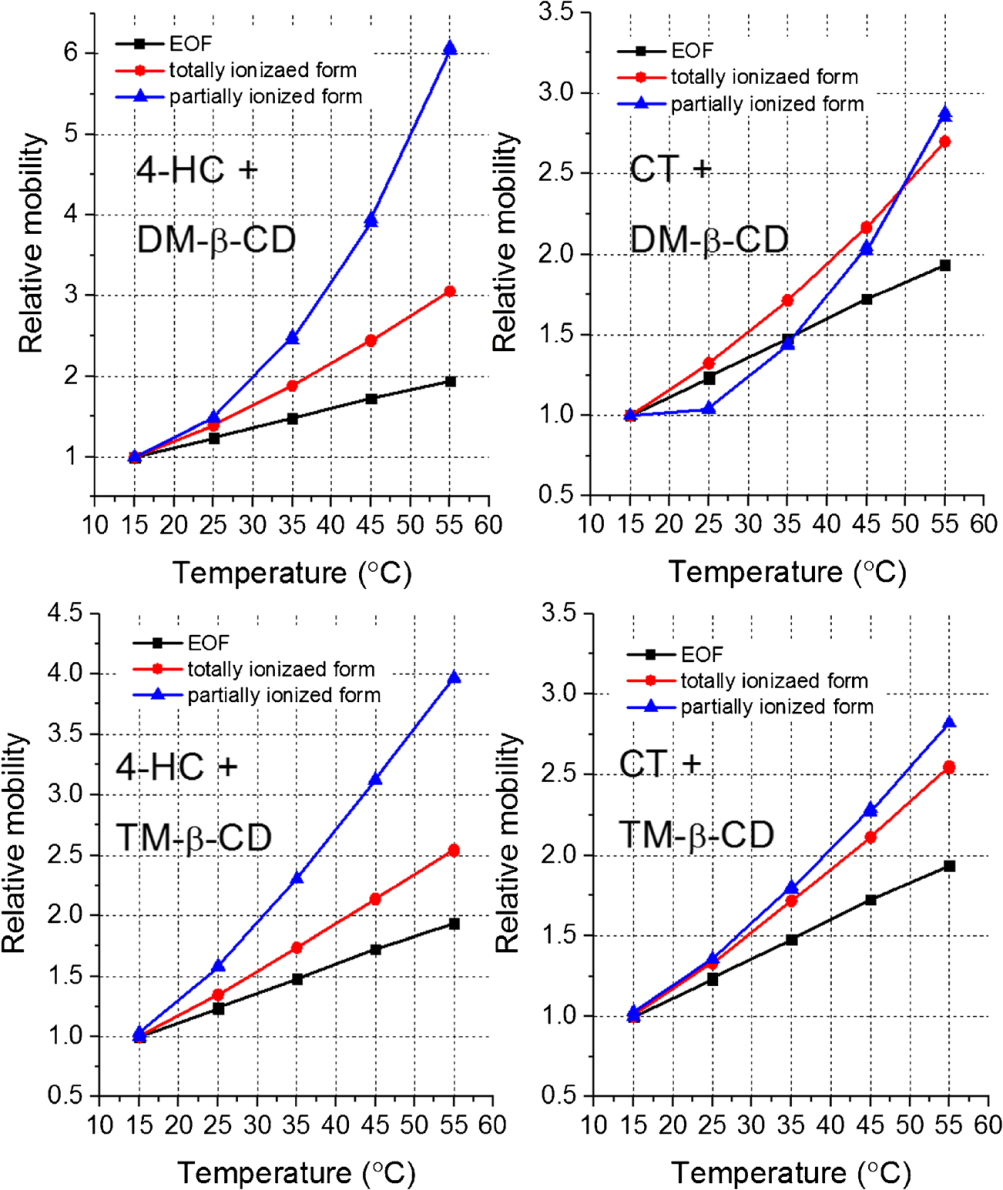
The plots of relative mobilities (divided by mobility at 15 °C): electroosmotic (*EOF*), electrophoretic obtained for the totally ionized form (pH 8.0) and for the partially ionized form (pH 4.6), versus nominal temperature of coolant

The p*K*_a_ values determined at various temperatures and the resulting enthalpic and entropic factors were compared between the reference host-free and two CD-containing systems (see Table 3). It turned out that the dissociation enthalpy and entropy change drastically upon the addition of both CDs, although the direction of this change is opposite for 4-HC and CT. For this reason, the magnitude of p*K*_a_ shifts drop with temperature for 4-HC and grow for CT. It is the sign that some thermodynamic effects accompanying interaction with CDs are different for these molecules. However, the observed p*K*_a_ values are only apparent ones because complexation degree is lower than 100%, and they cannot be assigned to the complex but only to a mixed population of the complexed and free analyte forms. On that account, the differences observed for 4-HC and CT may originate from two dynamic equilibria: protonation/deprotonation and complexation/dissociation of complex. A clear-cut interpretation of these results is difficult. The independent analysis of these two overlapped equilibria is nevertheless still possible for the neutral (non-ionizable) hosts without any additional experiments, although it has never been demonstrated so far. For this purpose, the plots of electrophoretic mobilities as a function of temperature are necessary, obtained for the EOF marker, totally ionized analyte, and partially ionized analyte. It is shown in Fig. 5.

**Table 3:** The values of acid dissociation constant (p*K*_a_), standard dissociation enthalpy (Δ*H*°), and standard dissociation entropy (Δ*S*°) determined without CD addition (host-free reference system) and in the presence of 10 mM DM-β-CD and TM-β-CD, with the respective differences (shifts)

Temp. (°C)	Host free	DM-β-CD		TM-β-CD	
	p*K*_a_	p*K*_a_	Shift	p*K*_a_	Shift
4-HC					
15	4.11 (0.03)	5.33 (0.02)	+1.22	4.77 (0.02)	+0.62
25	4.07 (0.02)	5.23 (0.02)	+1.12	4.64 (0.03)	+0.54
35	4.03 (0.04)	5.12 (0.04)	+1.05	4.54 (0.03)	+0.48
45	3.99 (0.03)	5.00 (0.03)	+0.98	4.46 (0.04)	+0.45
55	3.95 (0.04)	4.88 (0.04)	+0.89	4.38 (0.03)	+0.43
Δ*H*° (kJ mol^−1^)	7.4 (0.2)	20.4 (0.9)	+13.0	17.4 (0.7)	+10.0
Δ*S*°(J mol^−1^ K^−1^)	−6.4 (0.1)	−3.8 (0.4)	+2.6	−3.7 (0.3)	+2.7
CT					
15	4.46 (0.03)	5.21 (0.02)	+0.75	4.48 (0.03)	+0.02
25	4.32 (0.03)	5.18 (0.03)	+0.86	4.47 (0.02)	+0.15
35	4.20 (0.02)	5.15 (0.03)	+0.94	4.45 (0.03)	+0.26
45	4.09 (0.03)	5.08 (0.03)	+0.99	4.44 (0.03)	+0.36
55	3.98 (0.03)	5.01 (0.04)	+1.04	4.42 (0.02)	+0.45
Δ*H*° (kJ mol^−1^)	21.6 (0.1)	9.0 (1.3)	−12.6	2.7 (0.2)	−18.9
Δ*S*° (J mol^−1^ K^−1^)	−1.2 (0.1)	−8.3 (0.5)	−7.1	−9.2 (0.1)	−7.9

The values were determined using DCS (see the text for details). The values in brackets are the standard errors estimated during the function fitting step

To facilitate analysis, mobilities are calculated as the relative values, using the lowest temperature as a reference value, i.e., all values equal to 1.0 at 15 °C. The change of electroosmotic mobility with temperature stems mainly from a drop in viscosity. The mobility of the totally ionized form depends both on viscosity and complexation degree. The higher is complexation degree, the larger is hydrodynamic resistance during electromigration and lower mobility. As it is seen from Fig. 5, the relative mobility of the totally ionized analyte rises stronger than the relative mobility of the EOF marker; it is because temperature rise decreases complexation degree. In other words, complexation is exothermic and thereby the opposite process (complex dissociation) is enhanced by temperature elevation. This effect is very similar in each case, both for 4-HC and CT. The mobility of the partially ionized form, in turn, depends on viscosity, complexation degree, and ionization degree related to the p*K*_a_ value. In the case of 4-HC, it rises yet stronger than for the totally ionized form. This provides a proof that deprotonation, which entails molecule ionization and increase in mobility, is endothermic and thus facilitated upon temperature elevation. By contrast, for CT the relative mobilities of the totally and partially ionized forms change to a similar extent, what suggests that the ionization degree is rather constant and barely dependent on temperature. It shows that the differences in thermal dependency of p*K*_a_ shifts observed for 4-HC and CT are determined by the deprotonation-related interactions and weakly influenced by the complexation/dissociation equilibrium. Further discussion of these observations can be found in the next part of this manuscript (“Thermodynamic analysis”).

### Spectrophotometric CE-DAD method

Determination of p*K*_a_ shifts induced by the ionized host molecules, basing on measurement of electrophoretic mobility variation as a function of pH, is not straightforward. It is due to the fact that ionizations of the guest and the host influence each other, and a much more sophisticated model is required to estimate p*K*_a_ from a mobility-pH plot. The aim of this part was to show that, if the spectral properties of the analyte change upon ionization, the measurement of absorption at two properly selected wavelengths may be replace measurement of electrophoretic mobility. Accordingly, the *β* parameter is calculated as a function of pH.

9 $$ \beta =\frac{A_1}{A_1+{A}_2} $$

where *A*_1_ and *A*_2_ are the absorbance values measured at two different wavelength, in this case at 270 and 300 nm, respectively.

**Fig. 6:**
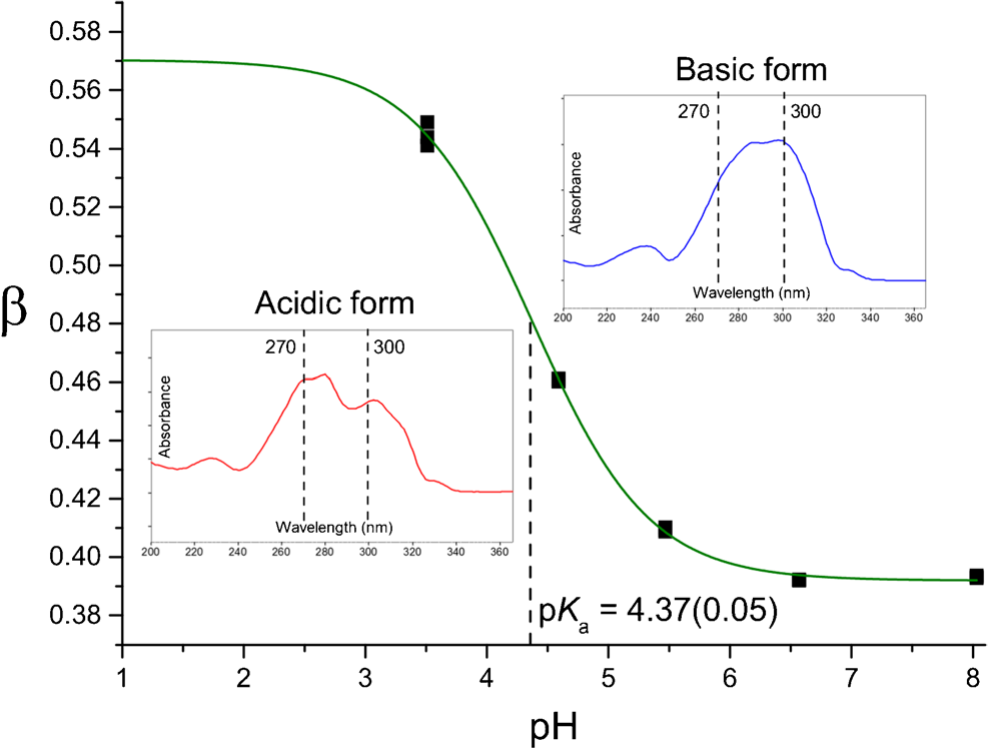
Illustration of the CE-DAD method used for determination of p*K*_a_ value of 4-HC in the presence of 4-sulfo-calix[4]arene; the *inset graphics* show the UV-vis spectra of the acidic (protonated) and basic (deprotonated) analyte forms; 270 and 300 nm are the wavelengths selected to determine variation of β value (see Eq. 9)

Such defined *β* changes with pH analogously to electrophoretic mobility and may be described by the same type of nonlinear function (Boltzmann sigmoid), with the p*K*_a_ value indicated by location of the inflection point. In addition, the values of concentration and molecular absorption coefficient are not needed, and this simplifies analysis. This CE-DAD was proposed by us in our recent work and used in determination of p*K*_a_ in the host-free system [12]. Herein, it was used to determine p*K*_a_ value of 4-HC in the presence of 5 mM 4-sulfo-calix[4]arene; it is illustrated in Fig. 6.

It was established that upon the addition of 4-sulfo-calix[4]arene, the apparent p*K*_a_ value increases from 4.07 to 4.37, what gives a positive shift in the order of +0.30 pH unit. This is the first p*K*_a_ shift observed for calixarene applied as a host with the use of CE-based methodology. Further studies embracing other molecules are planned for the future.

### Thermodynamic analysis

The values of p*K*_a_ shifts presented in Table 1 are positive for all CDs except with α-CD and 2HP-α-CD. However, the considerable negative shifts are observed only for CF, and they exceed −0.4 pH unit. In this particular case, complexation with the deprotonated (ionized) form is preferred over the protonated form. In all remaining cases, CDs exhibit stronger affinity for the protonated form of analytes which thereby is stabilized, and it is seen as the rise of p*K*_a_ upon the addition of host. A similar effect was observed in the past for other analytes [1], including different coumarin derivatives [7, 8]. It can be explained by the interactions between the hydrophobic guest molecule and the non-polar interior of CD’s cavity, which are stronger for the non-ionized and more hydrophobic form of guest.

The magnitude of p*K*_a_ shifts differ to some degree for four analytes (guests); however, the larger differences are noted for various CDs used as host (Table 1). The shift depends on cavity size, type of host substituent and even on substitution degree. The most interesting effect is observed for CDs characterized by the gradually increasing methylation degree. The p*K*_a_ shifts grow in the order: β-CD < Me-β-CD < DM-β-CD and drop significantly after addition of the third methyl group to the glucose unit—for TM-β-CD (for comparing the structures, see Fig. 2). It shows that there is a specific correlation between the structure of host, including its substitution pattern and the possible range of p*K*_a_ modification attainable on a given concentration level. The question however remains, what are the total p*K*_a_ shifts, i.e., the values measured in regard to the total complexation degree? It is possible that the different methylation level imposes significantly different complexation degree, and the total p*K*_a_ shifts of host-guest complexes are actually similar. The differences in electrophoretic mobility between the media containing DM-β-CD and TM-β-CD, seen in Fig. 3, support this concept. To answer this question, the further affinity studies are necessary, and they are beyond the scope of this work but they are planned for the near future as a natural continuation of this research path.

As it was mentioned before, the differences in the behavior of 4-HC and CT upon temperature increase follow most likely from the interactions specific for the deprotonation process. In general, the positive sign of Δ*H*° observed for the deprotonation (acid dissociation) equilibrium may be ascribed to breaking the chemical bonding with proton entailing the rise of the system heat capacity; in this case, the O–H interaction [5, 10]. Thus, the significant gain of Δ*H*° observed for 4-HC upon the addition of DM-β-CD and TM-β-CD (from around 7 to 20 and 17 kJ mol^−1^, respectively) may result from a specific host-guest interaction involving the protonated form of guest, broken upon deprotonation analogously as the O–H interaction. In the case of CT characterized by an opposite change of Δ*H*° after the addition of host, in contrast to 4-HC, some host-guest interaction could involve the deprotonated form of the guest and thus reduce an overall rise of heat capacity during deprotonation. One may also hypothesize that the additional aromatic ring in the structure of CT, absent in the structure of 4-HC (see Fig. 2), plays a key role in this effect. This outcome confirms that the thermodynamics of p*K*_a_ modification, including the changes of the enthalpic/entropic factors governing acid dissociation, may differ pronouncedly for the parent compound and its derivative.

## Conclusions

We showed how to enhance the analytical potential of CE as a tool for the cost- and time-effective screening/identification and accurate determination of the supramolecular p*K*_a_ shifts. The eCAP™ amine capillary ensures a repeatable determination of electrophoretic mobility values (RSD < 1.0%). The systematic errors caused by the voltage ramping and Joule heating effects can be examined and prevented by the double correction strategy (DCS). Both effects may compensate to some extent making the total p*K*_a_ error generally acceptable, below 0.05 pH unit. The use of DCS seems however advisable in other experimental setups, when the measured currents and the resulting temperature rise are high, and for a deep thermodynamic analysis comprising determination of dissociation enthalpy and entropy. The two-values method (TVM) requires measurement of only two electrophoretic mobility values to estimate p*K*_a_, it is a useful tool for a fast screening/identification and rough estimation of the supramolecular p*K*_a_ shifts. Plotting of the relative mobilities obtained for the EOF marker, totally ionized form, and partially ionized form against temperature allows one to compare thermal effects related to two distinct equilibria: deprotonation and complexation. The spectrophotometric CE-DAD method can be used in determination of p*K*_a_ shifts caused by the ionized host molecules, like sulfo-calixarenes. The magnitude of the apparent p*K*_a_ shifts and the corresponding thermal effects, like the change of the system heat capacity, may differ significantly for the structurally-related molecules. Providing an optimal host substitution pattern, in this case a double methylation of the glucose unit, was crucial for obtaining the maximal p*K*_a_ shift at a given concentration of host. Apart from the physicochemical studies, the methods and tips presented in this work may be very useful in development of advanced analytical methods using p*K*_a_ shifts to enhance resolution in the CE-based separations.

## Electronic supplementary material

ESM 1 (PDF 193 kb)
