# Supplementary material for: Enhancing effectiveness of capillary electrophoresis as an analytical tool in the supramolecular acidity modification
Source: Anal Bioanal Chem. 2017 Mar 24;409(14):3633–43. doi: 10.1007/s00216-017-0305-y (PMC5406438; doi:10.1007/s00216-017-0305-y)
Supplement: Supplementary file 1 — (PDF 193 kb) [file 216_2017_305_MOESM1_ESM.pdf]

Analytical and Bioanalytical Chemistry

Electronic Supplementary Material

Enhancing effectiveness of capillary electrophoresis as an analytical tool in the supramolecular acidity modification

Paweł Mateusz Nowak, Michał Woźniakiewicz, Magdalena Janus, Paweł Kościelniak

Composition of buffering solutions

Table S1 Composition of buffering solutions used in pK_a determination.

pH (measured)	Buffer type	Buffer ingredients	
3.51, 4.59, 5.47	Acetic buffer	CH_3COOH	CH_3COONa
6.57, 8.03	Phosphate buffer	NaH_2PO_4	Na_2HPO_4

The amounts of the given ingredients were calculated to ensure the constant ionic strength of 50 mM. The pH values presented in the table refer to the host-free solutions (without cyclodextrins and calixarene). For the host-containing buffers the exact pH values were measured prior to CE analysis.

Calculation of local temperatures by SUMET

According to the Krylov's method [1]:

$$\Delta T_{core} = \left(\frac{c\left(\frac{P_{av}}{L_{tot}}\right)^n}{g+\left(\frac{P_{eff}}{L_{tot}}\right)^n} \right) \cdot \left(\frac{P_{av}}{L_{tot}}\right) \qquad \text{(S1)}$$

$$\Delta T_{inlet} = ka^{\left(\frac{P_{av}}{L_{tot}}\right)} \cdot \left(\frac{P_{av}}{L_{tot}}\right) \qquad \text{(S2)}$$

where ΔT_{core} is the temperature rise in the thermostated capillary section, ΔT_{inlet} is the temperature rise in the non-thermostated section, P_{av} is the average electric power (W), L_{tot} is the total capillary length (0.60 m), while *c, n, g, k* and *a* are the parameters valid for the chosen capillary, in this case (50 µm i.d.): c = 6203, n = 0.147, g = 7608, k = 7.16 and a = 0.953 [1].

P_{av} was calculated as:

$$P_{av} = U_{nom}\left(\frac{t_{tot}-0.5t_{ramp}}{t_{tot}}\right) I_{exp}\left(\frac{t_{tot}-0.5t_{ramp}}{t_{tot}}\right) \qquad \text{(S3)}$$

where U_{nom} is the nominal separation voltage (30 kV); t_{ramp} is the ramp time, 0.17 min; t_{tot} is the migration time of a given analyte (for which electrophoretic mobility is calculated), I_{exp} is the current measured after the ramp time, at the 30th second of run.

The actual (effective) temperature values, obtained using Eq.8 (main text), are presented below:

Table S2 The nominal and actual temperature values obtained at various pH values (3.51 – 8.03, see Table S1)

nominal	pH 1	pH 2	pH 3	pH 4	pH 5
**15**	16.8	17.0	17.0	16.5	16.3
**25**	27.3	27.5	27.5	26.9	26.6
**35**	37.8	38.1	38.1	37.3	37.1
**45**	48.5	48.6	48.7	47.8	47.5
**55**	59.2	59.3	59.3	58.3	57.9

P_{av} was calculated in reference to the EOF marker (DMSO) in a host-free BGE.

A classical approach

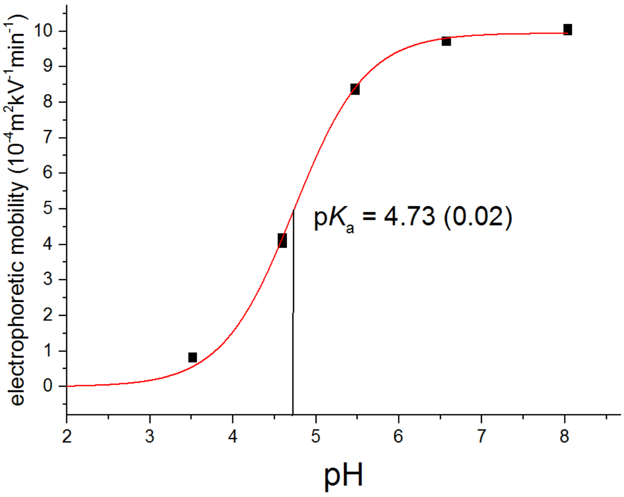

Fig.S1 Illustration of the classical method for determination of pK_a by CE, consisting in the measurement of electrophoretic mobility at various pH values and the nonlinear function fitting. The data obtained for 4-HC in the presence of 10 mM TM-β-CD, at 15°C

Calculation of *ΔH°* and *ΔS°*

The values of standard deprotonation enthalpy (ΔH°) and entropy (ΔS°) were calculated from the Van't Hoff model describing the relation between pK_a and temperature:

$$pK_a = \frac{\Delta H^\circ}{2.303RT} - \frac{\Delta S^\circ}{2.303R} \qquad \text{(S4)}$$

where R is the gas constant (8.3145 J·mol^{-1}·K^{-1}).

Accordingly, the pK_a values determined at various temperature were plotted against the inverse absolute temperature (1/T) and fitted by the linear function. Subsequently the enthalpic and entropic terms were calculated from the slope and intercept, respectively, see Fig.S2.

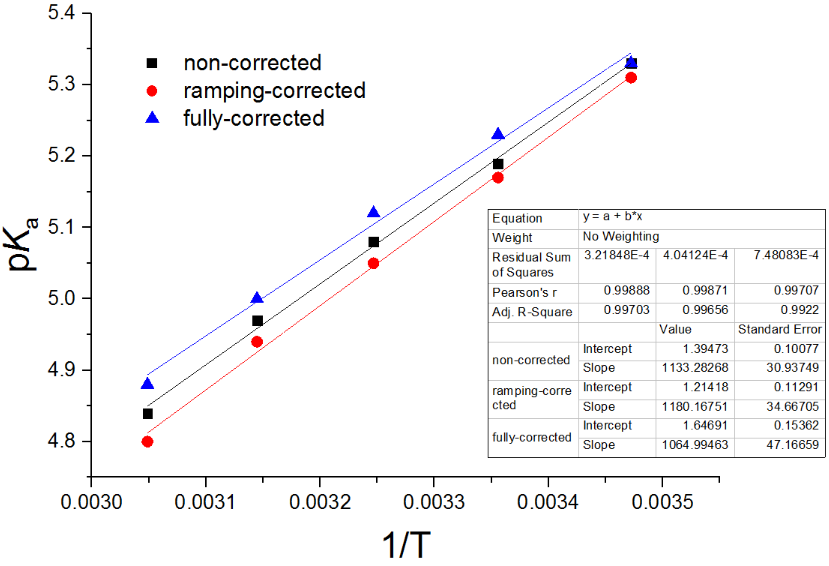

Fig.S2 The Van't Hoff plots obtained for 4-HC in the presence of 10 mM DM-β-CD, before and after two correction steps with DCS (see main text for details)

Repeatability of electrophoretic mobilities

In our recent work we postulated that the eCAP™ amine capillary is an optimal capillary type for determination of pK_a by CE owing to a great repeatability of electrophoretic mobility values, crucial in the minimization of the random errors [2]. In another work we showed that it also ensures a repeatable analysis in the micellar media [3]. In this work the amine capillary was for the first time applied to determine the supramolecular pK_a shifts. A simple test was performed. We estimated repeatability of electrophoretic mobilities of four analytes, expressed by the RSD (%) values, without and after the addition of 9 structurally diverse CDs. We also compared two pH values, where the analytes occur in the partially ionized (pH 4.6) and totally ionized (pH 8.0) forms. The results are shown in Table S3.

Table S3 The RSD (%) values (n = 3) obtained for electrophoretic mobility of four analytes (4-HC, 4,7-dHC, CT, CF) in BGE of two pH values, at the temperature of 25°C, in the various host-guest systems

System	4-HC	4,7-dHC	CT	CF	4-HC	4,7-dHC	CT	CF
	pH = 4.6				pH = 8.0			
reference	0.16	0.41	0.36	1.33	0.15	0.63	0.01	0.01
α-CD	0.12	0.45	0.35	0.45	0.08	0.51	0.04	0.27
β-CD	1.20	0.62	0.95	0.62	0.33	0.73	0.32	0.39
2-HP-α-CD	0.19	0.41	0.36	0.41	0.11	0.42	0.18	0.36
2-HP-β-CD	0.20	1.52	1.13	1.99	0.35	1.41	0.37	0.77
2-HP-γ-CD	0.73	1.07	0.47	0.47	0.17	0.29	0.10	0.14
2-HE-β-CD	0.47	0.11	0.98	0.69	0.08	0.57	0.06	0.37
Me-β-CD	0.50	0.49	1.66	0.49	0.22	1.08	0.04	0.10
DM-β-CD	0.81	0.52	0.43	0.52	0.48	0.35	0.49	1.81
TM-β-CD	0.15	0.22	0.70	0.22	0.34	0.72	0.27	0.23
average	**0.45**	**0.58**	**0.74**	**0.72**	**0.23**	**0.67**	**0.19**	**0.45**
median	**0.34**	**0.47**	**0.59**	**0.51**	**0.19**	**0.60**	**0.14**	**0.31**

reference – BGE without any supramolecular host

As it is seen the RSD values are very low, similarly for each analyte. Some differences between two pH values are noted, mostly in favor of the higher pH value. The repeatability is in general similarly good for the reference (host-free) and host-containing systems, the RSD values rarely surpass 1% for some CD-containing buffers. This confirms that the amine capillary exhibits a stable EOF and it is a suitable choice for the repeatable determination of electrophoretic mobility in various systems. It is especially important in regard to the media containing the macrocyclic molecules where electrophoretic mobility derives from various types of physicochemical interactions.

Another issue is that in the amine capillary the average migration times noted for the negatively ionized molecules are typically much shorter than in the uncoated bare silica capillary. It results from the stronger electroosmosis induced by the polyamine-coated inner capillary surface and its opposite direction, entailing migration of the negatively ionized analytes consistently with EOF direction. This enables their detection prior to the EOF marker, see Fig.3 in the main text. Therefore, the amine capillary may ensure both a very high repeatability and fast analysis of the acidic molecules.

References

[1] Evenhuis CJ, Musheev MU, Krylov SN. Universal Method for Determining Electrolyte Temperatures in Capillary Electrophoresis. Anal Chem. 2011;83:1808–1814.

[2] Nowak PM, Woźniakiewicz M, Piwowarska M, Kościelniak P. Determination of acid dissociation constant of 20 coumarin derivatives by capillary electrophoresis using the amine capillary and two different methodologies. J Chromatogr A. 2016;1446:149–157.

[3] Nowak PM, Woźniakiewicz M, Michalik M, Fiedor L, Kościelniak P. Nowak PM, Woźniakiewicz M, Michalik M, Fiedor L, Kościelniak P. Capillary coating as an important factor in optimization of the off-line and on-line MEKC assays of the highly hydrophobic enzyme chlorophyllase. Anal Bioanal Chem. 2017;409:1493–1501.
